# Enhancing Autonomy and Supervision During Psychiatry Residency: A Residents’ Perspective

**DOI:** 10.1192/j.eurpsy.2025.2375

**Published:** 2025-08-26

**Authors:** J. P. Carrasco, C. Conde-Pumpido, J. Esteve, B. Herraiz, J.-I. Etxeandia-Pradera, E. Aguilar

**Affiliations:** 1 Hospital Provincial Castellón, Castellón; 2 Hospital Clínico Universitario de Valencia, Valencia, Spain

## Abstract

**Introduction:**

The progressive acquisition of autonomy, balanced with adequate supervision, is essential during psychiatry residency. However, the adequacy of supervision and the smooth transition to autonomy remains a concern among residents. This study evaluates psychiatry residents’ perceptions of the current process of acquiring autonomy from the first to fourth year of training.

**Objectives:**

To assess the perceptions of psychiatry residents in Spain regarding the process of progressively acquiring autonomy and how supervision is managed throughout their residency.

**Methods:**

A qualitative analysis was conducted on responses to the survey question: “What should be improved in the progressive acquisition of autonomy and supervision from R1 to R4?” Data from free-text responses were coded thematically, with common themes identified and quantified.

**Results:**

Responses from 109 residents were analyzed. Thematic analysis revealed that 35% of residents emphasized the need for clearer and more structured feedback from supervisors, while 30% suggested more direct supervision during critical learning periods, particularly in the first two years. Additionally, 20% highlighted the inconsistency of supervision across different units, with some units providing much less oversight than others. Other suggestions included better scheduling of supervisory sessions (10%) and more frequent formal evaluations of their autonomy progression (5%).

**Table:** 

Theme	Percentage (%)
Structured feedback	35
Increased direct supervision	30
Consistency across units	20
Better supervisory scheduling	10
Formal evaluations of autonomy	5

**Conclusions:**

Residents identified several key areas for improvement in the process of acquiring autonomy, with a particular focus on the need for more structured feedback and increased supervision during the early years of training. Addressing these concerns may improve the overall quality of psychiatric education and resident preparedness.

**Disclosure of Interest:**

None Declared

